# Is it morally permissible for general practitioners to disclose their opinion on a woman’s decision on abortion?

**DOI:** 10.1186/s12910-020-0464-9

**Published:** 2020-03-12

**Authors:** Lynnlette Aung, Selena Knight

**Affiliations:** 1 St George’s University, University of London, London, UK; 2 King’s College London, London, UK

**Keywords:** Ethics, Clinical ethics, women’s health, Professionalism, Abortion

## Abstract

**Background:**

This paper considers ethical dilemmas arising where a patient asks their General Practitioner for advice and their personal opinion regarding whether or not to have an abortion. Patients often seek their General Practitioner’s advice regarding treatments and procedures, which may occasionally lead to the General Practitioner facing a difficult dilemma of whether to share their personal opinion with their patient. As General Practitioners are more accessible as the first point of contact for patients and often have a closer relationship with them, they may be particularly exposed to such situations. Additionally, the significance of abortion as a sensitive topic and the fact the General Practitioner may have their own personal viewpoint on its morality may make it particularly difficult for them to know how to respond to such a request.

**Main text:**

This paper explores the difficulties arising in such a situation and considers whether it could ever be ethically justifiable for General Practitioners to express their opinions on such a matter. We consider the duties of a doctor, and highlight the need for clearer guidance for healthcare professionals on managing tensions in their professional boundaries between their personal moral views and their professional responsibilities. A range of ethical viewpoints are considered to explore how a doctor might ap, in particular the principle of autonomy, virtue ethics, and consequentialism.

**Conclusions:**

This article recognises that a General Practitioner in a situation such as this faces many ethical challenges. We propose that offering their opinion to the patient where specifically requested may be morally justifiable. A virtue ethics approach in particular requires that the General Practitioner applies practical wisdom to make this decision, and where they do disclose their opinion ensure this is done so in such a manner that it does not harm the patient and promotes flourishing. We encourage GPs and other healthcare professionals to consider their own moral perspectives on sensitive issues such as abortion, and reflect on how their moral viewpoints have the potential to influence their practice. In doing so, we hope clinicians can be better should they be faced with a situation such as this.

## Background

The Abortion Act 1967 is the legal statute governing abortion in England, Scotland and Wales, outlining criterion which must be satisfied to legally terminate a pregnancy [1]. Two clinicians must confirm that the relevant criterion have been met [1]. Whilst this would not commonly be a General Practitioner (GP), it is not unusual for them to be the patient’s first point of contact when considering this option, and so the GP may have a role in signposting or referring them to abortion services.

Regardless of the circumstances of the patient or consultation, part of a GP’s role is to provide patients with appropriate information to empower them to make informed decisions. Where a patient consults their GP wishing to discuss abortion, the GP would be expected to provide broad information regarding the clinical risks and benefits of the procedure, but may also feel it appropriate to provide other information that may be relevant for the patient such as the potential emotional implications and long-term medical and psychological sequalae. GPs would not be required to provide detailed clinical or technical information, which is more commonly provided by a dedicated counselling service or the abortion provider themselves. Given that the requirements in terms of appropriate information provision are not in dispute, this article focuses on the ethical challenges arising in terms of whether the GP should share their personal opinion about whether the patient should have an abortion, where such opinion has been specifically sought by the patient.

Although abortion itself is a of course a highly morally sensitive subject, this article focuses on the ethical challenges arising out of the patient’s specific request for the GP’s advice and the relationship between the patient and GP, rather than the morality of abortion in itself. Irrespective of whether the patient or GP feels abortion is morally right or wrong, the issues discussed here are not *whether* it is right or wrong, but rather, irrespective of their opinion, whether that opinion should be shared with the patient. This article only considers circumstances where a GP has specifically been asked by the patient for their opinion, not situations of spontaneous disclosure decided by the GP, which generally be considered unprofessional and not ethically appropriate.

In order to illustrate the ethical challenges arising from such a situation, the following hypothetical case of Mrs. X shall be considered:

*Mrs X is 34 year old woman who is 9 weeks pregnant and consults her GP, whom she knows well and has a longstanding therapeutic doctor-patient relationship. She is unsure whether she wishes to continue her pregnancy. She has a history of severe anxiety, and explains that she worries she would be unable to commit to looking after a child, and that whilst she is currently stable her anxiety could relapse. She is happily married and would like children in the future, but fears now is not the right time for a pregnancy. She is visibly distressed about what to do. She appreciates the emotional implications of having an abortion, and wonders if she would regret it. She asks the GP for their personal opinion, specifically what her GP feels she ought to do and what they would do in her position.*

Vignettes such as this have been used to illustrate ethical dilemmas which can arise in the context of the doctor-patient relationship. Of comparison, Toon discusses the case of a doctor who is asked to advise couple of have recently had a baby with severe anoxic brain damage [2].. In the case the doctor strongly advises the couple to consider adoption. Toon suggests that the doctor here is taking the place of a wise friend, a concept we shall similarly apply to the case of Mrs. X. Whilst Toon’s case might raise controversy around the actual decision which is made by the advising doctor (that due to the baby’s disability they ought to be adopted), the case of Mrs. X does not consider the content of the decision or GP’s opinion, but rather whether this should even be shared in the first place.

With reference to the case of Mrs. X, this article considers a number of perspectives to explore these ethical issues. Firstly, the professional duties of a doctor are considered with specific reference to the UK’s professional guidance issued by the General Medical Council (GMC) [3]. The principle of autonomy is then considered, in particular how the GP’s decision of whether or not to disclose their opinion may subsequently influence the patient and either promote or limit her autonomy. We then discuss the nature of the difficulties arising from deciding where to draw and how to define professional boundaries relating to the GP’s professional duties and their personal moral opinion. Following this, a virtue ethics approach is considered, with particular reference to the concept of the GP acting as a “wise friend” in this situation. We consider how this concept can be both inherently challenging but also beneficial in helping the GP decide what to do. We also consider the need for the GP to apply practical wisdom if they inadvertently take on this role, and discuss what different forms of wisdom this might encompass. Other aspects of virtue ethics such as virtuous character traits and the concept of flourishing are discussed. Finally, a consequentialist approach is considered, where the complexities in predicting the consequences of decisions surrounding abortion and disclosure of opinion are highlighted.

This paper focuses on GPs for a number of reasons. GPs are particularly well placed to provide continuity of care, and the longer-term relationships can give them a better understanding of their patient’s circumstances compared to those in secondary care [4]. Patients may also be more inclined to seek advice from their GP about personal or sensitive matters, particularly where they have an especially close or trustworthy professional relationship, and are easily accessible as a first point of contact. That said, due to the ability of patients to self-refer directly to abortion services, GPs may not have these conversations often, and so whilst patients might expect their GPs to have the knowledge and skills to have such conversations, a lack of experience and confidence may mean this is in fact not the case. Additionally, the highly morally sensitive nature of abortion means that the GP may have their own views on its morality, although they may not have considered how these could affect their patient consultations until unexpectedly faced with a situation such as that described here. It is therefore particularly important that GPs consider such a situation in advance so they have an approach which they feel comfortable with and is right for both themselves and their patient.

Whilst this article focuses on GPs, the issues discussed here are relevant to other doctors and healthcare professionals, who will invariably also face challenging decisions arising from the interface between their professional role and their personal moral viewpoints. Additionally, whilst this article focuses on the example of abortion, the application of the ethical principles and issues discussed have relevance to situations involving other sensitive procedures or decisions.

## Main text

### The duties of a doctor

In the UK, the professional duties of a doctor are outlined in the GMC’s ‘Good Medical Practice’, to which all UK doctors must abide [3]. The GMC states that to provide an appropriate standard of care doctors should consider “psychological, spiritual, social and cultural factors” (p.7) [3]. Doctors are expected to care for patients holistically, which includes attempting to understand their personal situation and values.

The GMC offers guidance to doctors in terms of disclosing personal beliefs, clearly advising: “you must not express your personal beliefs (including political, religious and moral beliefs) to patients in ways that exploit their vulnerability or are likely to cause them distress” (p.18) [3]. This recognises the potential risk of causing offense, distress, or unduly influencing the patient which may result from disclosing their personal view about a patient’s situation. This is particularly pertinent in a case such as that of Mrs. X, given the sensitive and life-changing nature of the decision in hand.

Separate guidance is offered in the GMC’s guidance ‘Personal beliefs and medical practice 2013’, which states that doctors are allowed to express their own personal beliefs “only if a patient asks [the doctor] directly about them, or indicates they would welcome such a discussion” (p.5) [5]. This suggests it may be permissible for the GP to disclose their opinion to the patient in this scenario, given that they have specifically sought it. Disclosing their opinion might be further supported by the GMC’s requirement for doctors to act with “honesty and integrity” (p.21), and to “respond honestly to [patients’] questions” (p.13) [3].

Whilst this professional guidance endeavours to provide clarity, there is the potential for these professional duties to conflict. If the GP discloses their opinion at the patient’s request, whilst they are being honest, there is a risk it might exacerbate her distress if it differs from the option she was perhaps considering, and particularly given it might be difficult to predict how she might respond to whatever the GP’s opinion is. Conversely, if they decline to share their opinion to minimise any potential distress, the patient may perceive them as failing to fulfil their duty to act with honesty and integrity. Whilst these duties and professional guidelines are intended to provide guidance on such circumstances, they do not necessarily help the GP in making a decision in a case such as this.

### Autonomy

Respect for autonomy, described as “deliberated self-rule” (p. 184) is a founding principle of healthcare ethics [6]. It is often considered a cornerstone of a therapeutic doctor-patient relationship which should be promoted wherever possible. Significantly interfering with or influencing a patient’s decision risks that such a decision is not truly autonomous and undermines the patient’s free will [7]. Mrs. X’s autonomy must be carefully considered, as there is a chance that the GP’s opinion may influence her decision.

Nevertheless, it has been proposed that respecting a person’s autonomy does not necessarily mean non-interference altogether [4]. Part of a doctor’s role is to enable patients to exercise their autonomy, with one part of this being the provision of necessary and relevant information to allow them to make an informed decision. In Mrs. X’s case, this might include the GP sharing their opinion on the matter, given that they have specifically been asked. Furthermore, it is Mrs. X’s own choice to ask her GP for their opinion and so arguably in doing so they are acting according to the her wishes and thus respecting their autonomy.

The principle of respect for autonomy conveys that one should be free from coercion in decision-making [7]. Considering the case of Mrs. X, the GP disclosing their opinion would not necessarily equate to coercion – this term portrays a patient being forced into a decision against their will, whereas here the GP has been invited to share their opinion by the patient. There are measures which the GP might take to ensure that in sharing their personal opinion they do not risk impeding a patient’s autonomy nor coerce the patient, for example reiterating to the patient that the final decision is theirs to make, or even suggesting to the patient before providing their opinion that it may influence their final decision.

Whilst sharing their personal opinion could be considered to have the potential to impede Mrs. X’s ability to make an autonomous decision, if disclosed in an appropriate and sensitive manner it could be ethically justified for the GP to do so. It may even promote her autonomy as provision of information can supporting her in more autonomous and better informed decision-making. Clearly the manner by which the GP does so is crucially important to ensure Mrs. X continues to be able to exert her autonomy, without undue pressure, by making the final decision herself.

### Professional boundaries – where do we draw the line?

The GMC highlights that appropriate professional boundaries “are essential to maintaining a relationship of trust between a doctor and a patient” (p.5) and to ensure professionalism is upheld in practice [5]. Dilemmas arise when deciding where to draw the lines of professional boundaries in medicine, particularly in general practice. This reflects the fact that GPs may take on multiple roles in order to provide a holistic approach to patient care, and that they may have longer-term relationships with their patients [4]. Patients may consider their GP to be their doctor, but also to potentially fulfil other roles including confidant, professional, advice-giver, advocate, and in some circumstances, even friend. The type of relationship that forms between the GP and the patient will influence the way in which professional boundaries are established and experienced by both parties. A number of different models of physician-patient relationship have been described – paternalistic (the physician determines what intervention is best for the patient); informative (the physician provides the patient with information and the patient selects the option they prefer); interpretive (the physician elicits the patient’s values and wishes and helps them determine which option best achieves these); and finally deliberative (the physician helps the patient choose the best values which can be realised in the clinical situation) [8]. The challenge faced by the GP in Mrs. X’s situation is how they can attempt to continue the already established therapeutic and supportive relationship they have with Mrs. X, whilst still maintaining their professional integrity and appropriate professional boundaries.

In the case of Mrs. X, the GP is being asked for their personal opinion but in a professional capacity, indicating potentially blurring of the boundaries which normally exist between a patient and their doctor. Such a request might indicate that she views her GP more akin to a “wise friend” than a medical professional, a status which has been described by Toon [4]. This might be more likely to occur where a GP plays a significant role in helping a patient with more personal or sensitive issues such as mental illness or supporting with psychosocial difficulties, or where there is a pre-existing long-term therapeutic relationship. Such a relationship may represent either the interpretive or the deliberative model – the patient is not purely asking for scientific advice or direction from the GP, but rather is personally asking for advice and thus inherently the personal values of both the GP and the patient may be shared. It has been suggested that whilst boundaries are important to preserve objectivity, “on occasions, it feels the right thing to do to break down those boundaries just enough to help the patient over a hurdle.” [9] Neighbour argues that doctors have an authority that they can use in the patient’s best interest, called the “apostolic function” [9]. If one views medical authority as something to be used for the patient’s good, then it may be appropriate for a GP to assist a patient’s decision-making by offering advice, assuming such advice is in the patient’s best interest [9].

However, taking on the role of “wise friend” has the potential to be problematic*.* In most circumstances it is not considered appropriate for a doctor to befriend their patient in the true sense of the word, and particularly here where Mrs. X is vulnerable and seeking their doctors advice on a highly morally sensitive issue. There is also a lack of reciprocity in the relationship which might normally be expected from a true friendship - whilst Mrs. X has sought her GP’s opinion and advice, the GP would not reciprocate this and seek Mrs. X’s advice were they to be faced with a difficult decision themselves. Furthermore, the concept of friendship in this relationship is problematic because of the significant power imbalance which inherently exists between doctors and their patients. Doctors possess a certain power by virtue of their professional role, which whilst can be beneficial in some situations, also has the potential to place the patient at risk of harm. This would be of particular concern in Mrs. X’s case where she is particularly vulnerable due to her situation and the sensitive nature of the issue in question. If the power imbalance is significant, there is risk that the patient may feel unable to refuse the advice or feel unduly influenced by it, impeding on her autonomy. It is imperative that the GP is aware of the power differential existing between themselves and Mrs. X and how this might impact on the consultation. It may be necessary for them to adapt their consultation style and carefully consider what information they provide to the patient (and how they provide it) to manage this power imbalance and avoid any potential adverse outcomes which might arise.

Due to the sensitivity of abortion, it is highly possible the GP will have their own personal moral viewpoint about this topic and potentially specifically what Mrs. X should do. Depending on a number of factors (e.g. the strength of the GP’s views on the morality of abortion; the patient’s own views regarding abortion; the exact circumstances of the patient and the reason for their request for an abortion; any prior experiences of the GP), a consultation such as this might evoke certain and potentially difficult emotions for the GP. This is not necessarily problematic - it has been proposed that doctors function better and patients experience a better service where doctors have few boundaries between their personal and professional self, which could involve sharing their emotions and moral viewpoints [10]. Acknowledging and sharing emotions has the potential to strengthen the doctor-patient relationship - the act of a doctor disclosing their emotions has been proposed as enabling patients to view them as a “fellow human”, with positive implications for the relationship [11]. Emotions and conscience have been considered important and valid features of British general practice [12] which have the potential to be used constructively to contribute to an empathetic approach to patient care.

However, safeguards are necessary to ensure that the GP manages these emotions and any potential disclosure of their opinion is done so appropriately to ensure no harm is caused. Doctors should be aware of their emotions and consider how these might influence the consultation, both consciously (for example when they are deciding what they should advise the patient and how their emotions might influence this), but also subconsciously (for example ensuring their tone does not appear judgmental, or even considering aspects of the consultation such as their body language). Self-awareness is an important component of professionalism and particular relevant in a case such as this. As Papanikitas describes, whilst GPs do not necessarily operate in a neutral state, they must remain aware of their values and consider the extent to which such values and beliefs should be allowed to influence their practice [13].

### Practical wisdom, virtues and flourishing

Virtue ethics is implicit in much of the literature in medical professionalism, whereby doctors are encouraged to demonstrate traditional virtues and positive character traits in order to care for their patients in accordance with moral principles. Virtue ethics has been suggested to promote a more holistic ethical approach, as it often involves consideration of wider aspects of the patient’s life beyond just their medical needs [14]. This holistic approach to patient care is particularly advocated in general practice, and thus virtue ethics has particular relevance to the case of Mrs. X.

A central component of virtue ethics is that one should use practical wisdom to choose the morally right course of action. Toon describes practical wisdom as “an ability to perceive situations from a virtuous perspective and to analyse the virtuous course of action” [2].. There are a number of forms of “wisdom” which might need to be drawn upon if the GP is to use practical wisdom to choose the right course of action in the case of Mrs. X.

Firstly, the GP might be expected to have sufficient knowledge (‘wisdom’) of the patient and their circumstances, values, desires, and personal narrative to be able to decide whether it is appropriate in the first place to share their opinion with the patient. For example, if the GP had a certain opinion that they knew directly opposed the views of the patient (for example because of the patient’s religious views) they might be more wary of sharing it in order to avoid causing undue distress or conflict. Similarly, if the GP knew the patient was particularly vulnerable and influenceable they might be less inclined to share their opinion to ensure they do not unduly influence the patient. However, it is of course difficult for a GP to have sufficient in depth knowledge of a patient’s personal circumstances to be able to really understand and advise them. Even where doctors and patients have a close relationship, they would be unlikely to share their true deep inner thoughts with their doctor, thus limiting the knowledge the GP would be able to have in this area.

Another form of ‘wisdom’ the GP might be expected to have would be about abortion itself. Mrs. X might understandably expect that their GP would have prior experience in relation to abortion through their professional background - for example that they may have previously advised women in similar circumstances, or have seen the consequences of women choosing different options when faced with a similar decision. Mrs. X may perceive that such experience would place her GP in a position to provide accurate and beneficial advice and recommend the right course of action. However, as already discussed, many women refer themselves directly to abortion services bypassing their GP, and much of the counselling surrounding abortion is provided by dedicated abortion services. The GP may therefore have limited experience in advising women in Mrs. X’s situation, and so may lack the knowledge and experience to be considered *wise* in this sense. Additionally, given that every woman’s circumstances are different, even where a GP may have experience with patients who undergone abortions (with either positive or negative outcomes), it would not be possible for them to these experiences to be able to provide advice regarding Mrs. X’s specific circumstances.

In order to apply practical wisdom, virtue ethics also the moral agent to act according to virtuous characteristics and behaviours. It is therefore necessary to consider which virtues the GP might apply in this situation in order to choose the morally right course of action. Whilst there is no set list of virtues, there might be certain ones which are of particular relevance here. On one hand, the virtuous characteristics of integrity and honesty might be used to justify the GP disclosing their opinion, particularly given that Mrs. X has specifically requested it, and so if the GP has a particular opinion some may consider it dishonest and disingenuous to not share it. However, frank disclosure of the doctor’s opinion might oppose other virtues such as compassion (for example if the disclosure is likely to cause the patient distress), or discernment. Aristotle proposes virtues as traits lying in the middle between two vices [15], and frank disclosure with no thought to the consequences, whilst being honest would oppose other virtues and not be considered to in the middle of such vices. Applying virtues therefore cannot necessarily direct the GP to whether they should or should not disclose their opinion, but it does encourage them to reflect on the manner by which they might make and communicate their decision, whether this be disclosure or non-disclosure.

Finally, where considering a virtue ethics approach to a situation such as this it is necessary to consider the concept of flourishing, which forms an important aspect of this moral theory. It has been suggested that “the main purpose of medicine is to help patients construct a flourishing narrative” (p.45) and therefore the most virtuous course of action would be that by which the agent uses appropriately chosen virtues to maximise Mrs. X’s flourishing [14]. Given that she has asked the GP for their opinion to help her reach a decision, one might argue that the GP is obliged share this to help her reach an informed decision which is most likely to lead to her achieving eudaimonia and a flourishing narrative. However, difficulties may arise in determining the ideal flourishing narrative for the patient, as the GP’s idea of what is best for the patient or what may constitute a flourishing narrative may differ from the patient’s. Additionally, should the GP, even unintentionally, expresses their opinion insensitively they may cause the patient undue distress.

By using virtues to guide actions, we can attempt to resolve some of the ethical dilemmas encountered in medicine. In this case, a virtue ethics approach requires the GP to consider which virtues might guide them to make the right decision, and how such decisions should be shared with the patient. Perhaps more helpfully it also encourages the doctor to employ practical wisdom, considering the patient, their circumstances, the procedure in question, and also their own prior knowledge and experience. Disclosing their opinion, if this is the course of action taken by the GP, must be undertaken using the GP’s practical wisdom to do so with care and awareness for the patient’s circumstances to minimise any potential distress and avoid demonstrating vices. Through employing knowledge, experience and practical wisdom, provided the GP shares their opinion in a sensitive manner, they may be able to offer important and helpful advice which the patient may wish to consider in their decision-making.

### Consequentialism

Consequentialism claims that the “moral rightness of acts… depends only on the consequences of that act.” [16] Therefore a consequentialist would deem the ethical course of action as being the one which promotes the best consequences for Mrs. X.

Sharing their opinion with Mrs. X, having been asked, could be a justified course of action for the GP from a consequentialist perspective, as doing so may build trust and in the long-term be beneficial for the doctor-patient relationship [17]. This might be particularly important for a patient such as Mrs. X, who’s anxiety may mean that she requires an ongoing supportive relationship with healthcare professionals.

However, there are also potential negative consequences of the GP sharing their opinion with Mrs. X. Establishing which course of action results in the best overall consequences is difficult, and would be particularly so here. It is extremely challenging, arguably impossible, for the GP to determine whether continuing the pregnancy or having an abortion would result in the best consequences for her. Abortions are often considered only in the short-term sequelae, resulting in an inherent difficulty in taking a consequentialist approach to such cases. If the GP offers their opinion, whatever it may be, and the patient disagrees or is distressed by their view, or takes their advice and later regrets their decision, it could have devastating consequences personally, professionally, and potentially even medico-legally.

It is extremely difficult for a consequentialist approach to be used to recommend a course of action in this case. Given the significant uncertainty surrounding whether an abortion or continuing the pregnancy would result in the best overall consequences, it is extremely difficult to predict the consequences of the GP offering their personal opinion. Whilst on one hand a consequentialist approach may support disclosure in order to foster a positive doctor-patient relationship, doing so could also hinder or adversely impact the patient’s longer-term independence in decision-making. Additionally, there is potential for the patient to blame the GP for their final decision, having been advised, if adverse events occur post decision-making.

## Conclusions

This article has explored a number of perspectives to consider if it can be appropriate for GPs to offer their personal advice to a patient about whether they should have an abortion.

From a professional perspective, the GMC guidelines do not necessarily provide clarity in this situation, describing potentially conflicting duties, stating that personal beliefs can be disclosed when specifically requested but not if it causes the patient distress, the chance of which may be hard to determine in cases such as this. The GMC also requires doctors to act with honesty and integrity, which can be problematic when taken alongside their guidelines stating not to express personal beliefs. As a result, the GMC guidelines may not always fully equip doctors in knowing how to approach situations such as that in the case of Mrs. X, leaving GPs to their own ethical judgements.

Neutrality is underlined in the GMC guidelines, and seems to serve this purpose. However, they also emphasise doctors working towards their patient’s best interests which is difficult if GPs are focusing on being neutral and protecting themselves medicolegally. This is perhaps a reason for the GMC to update their guidance on this matter to provide more clarity to enable doctors to make appropriate decisions in such circumstances. There may also be a need for training for medical students, doctors and other healthcare professionals on how to approach such situations where they might be asked disclose their personal opinions in emotive situations.

The case here presents the GP with many ethical challenges – whether they should disclose their opinion, if so how it should be done, as well as of course the inherent moral difficulty which arises from questions on the topic of abortion. The GP has been placed in a difficult situation, as the request for their personal advice would generally be considered to fall outside the remit of a doctor’s traditional role, blurring the professional boundaries which form part of a traditional doctor-patient relationship. The GP may have their own personal opinions regarding the morality of abortion, and it may be difficult for them to know the extent to which these opinions should be shared with the patient, if at all. If doctors have particularly strong opinions, it may be difficult for them to remain neutral [18]. It is therefore essential that GPs and other healthcare professionals are aware of their pre-existing moral viewpoints and emotions to ensure they do not adversely impact the patient or consultation. They may also need to be aware of the potential medicolegal ramifications of providing advice, for example should the GP advise a specific course of action and the patient feel they are harmed, should they follow their advice.

In offering their personal opinion to the patient, there could be some potential positive value. Given the patient has specifically requested her GP to disclose their opinion on her pregnancy, this may be indicative of an established and trustworthy relationship where such advice is valued and welcomed by the patient. It may also promote her autonomy in helping her reach an informed decision provided disclosure is managed sensitively and appropriately (for example the doctor ensuring they provide balanced information, offer their opinion in a non-judgmental way and emphasise that the final decision is of course the patient’s). It is essential that a GP apply practical wisdom to this situation – whilst they not be able to acquire a full understanding of all of these, they should endeavour to try to understand to the best of their abilities the patient’s circumstances, have some knowledge of abortion and its consequences, and of course have awareness of their own prior experiences, emotions and moral viewpoints to try to address the patient’s request in a constructive way to avoid harm. Ultimately, the nature of the decision in question as being extremely morally sensitive means that not only is the GP in this circumstance facing an ethical dilemma, but the patient also is. Simply acknowledging the presence of this moral dilemma may be an important start to any subsequent conversations between the patient and the doctor about what they should do. Situations such as this give rise to difficult decisions for both the patient and doctor, and whilst there may not be one size fits all solution, recognition of the challenges of a decision such as this and the potential impact on the doctor-patient relationship are essential.

## Data Availability

Not applicable.

## Abbreviations

GP: General practitioner
GMC: General medical council
